# Evaluation of the mechanical stability of intraocular lenses using digital image correlation

**DOI:** 10.1038/s41598-023-36694-0

**Published:** 2023-06-09

**Authors:** Iulen Cabeza-Gil, Javier Frechilla, Begoña Calvo

**Affiliations:** 1 Aragón Institute of Engineering Research (i3A), University of Zaragoza, Zaragoza, Spain; 2 Biomaterials and Nanomedicine Networking Biomedical Research Centre (CIBER-BBN), Zaragoza, Spain

**Keywords:** Biomedical engineering, Mechanical engineering, Translational research

## Abstract

This study aimed to evaluate the mechanical stability of seven different intraocular lens (IOL) haptic designs by using digital image correlation to measure their mechanical biomarkers (axial displacement, tilt, and rotation) under quasi-static compression. The IOLs were compressed between two clamps from 11.00 up to 9.50 mm whilst a 3D deformation dataset was acquired every 0.04 mm. Results revealed that flexible and mixed IOL designs exhibited better mechanical response for smaller compression diameters compared to stiff designs. Conversely, stiff designs performed better for larger compression diameters. These findings may aid in the selection and development of more mechanically stable IOL designs.

## Introduction

**Figure 1:**
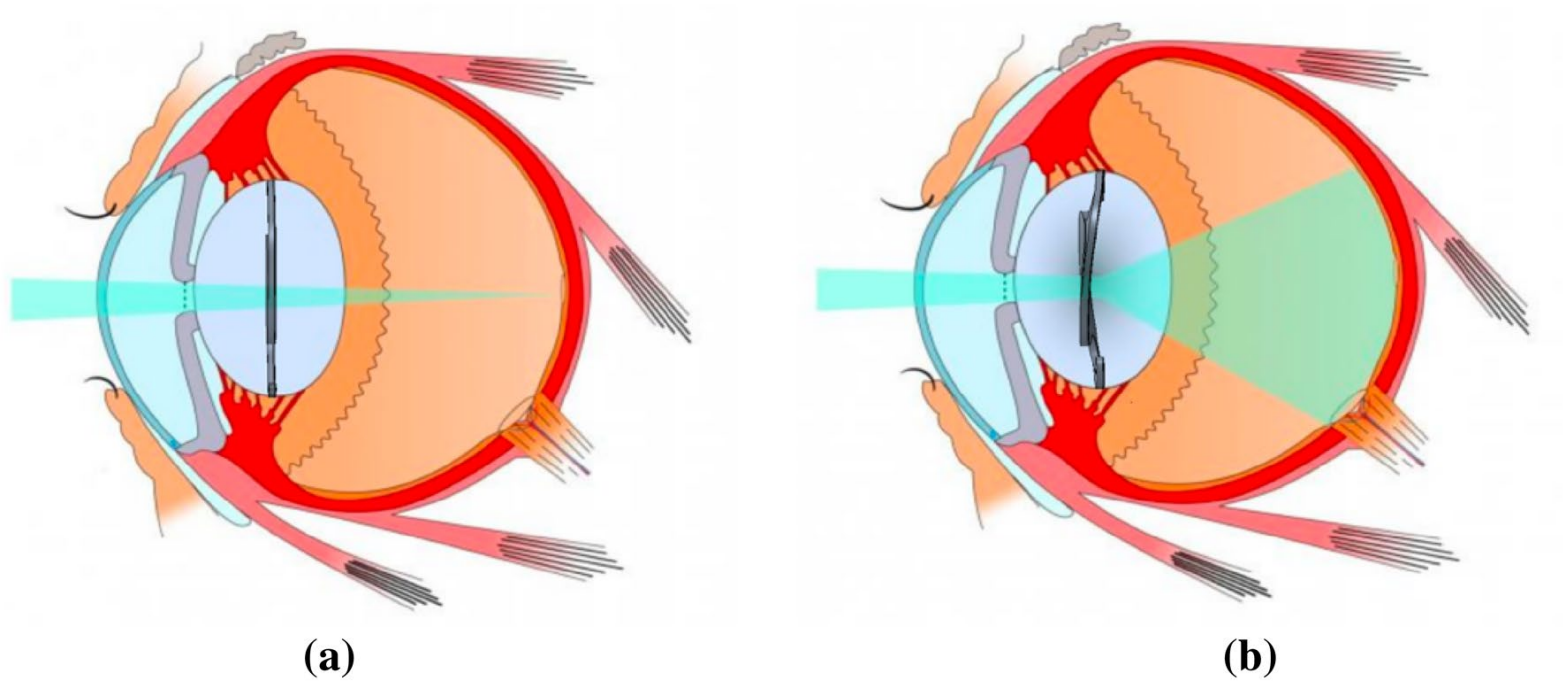
Outline of the effect of the mechanical stability of the IOL in the optical performance.

The position of the intraocular lens (IOL) inside the capsular bag is critical for the visual performance of the patient after cataract surgery^1,2^. Intraocular lens axial displacement can lead to residual refractive error^3,4^; tilt can require explanation or repositioning^5,6^; while rotation and decentration are crucial factors for toric and asymmetrical multifocal IOLs^7–9^, see Fig. 1.

To ensure the mechanical stability of IOLs before commercialization, IOLs are tested according to the ISO 11979:3:2012—*Mechanical properties and test methods*^10^, which consists in compressing the IOL in a single-size compression well of 10.00 mm and evaluating the main mechanical biomarkers (axial displacement, tilt, rotation and decentration), which are related to the optical performance of the patient postsurgery^11^. These mechanical biomarkers are usually measured manually^3,4,11–14^, involving both measurement error due to the difficulty of measuring at the micro-scale and significant time cost.

The ISO 11979–3:2012 standard is also used to determine whether modifications to existing models require clinical investigations. However, one limitation of the ISO is that the IOLs intended for the capsular bag must be measured in a compression well with a diameter of 10.00 mm, when the variability of the postcataract capsular bag diameter is relatively higher^15–18^.

To account for variability of the postcataract capsular bag and address the tedious work of manual measurement, this study aimed to evaluate seven different IOLs in a quasi-static compression test, from diameter of 11.00 to 9.50 mm with an automatic measuring method. The study measured the mechanical biomarkers of these IOLs using an automatic measuring method called Digital Image Correlation (DIC)^19,20^, while varying the diameter of the compression well from 11.00 to 9.50 mm.

## Methods

DIC is a non-interferometric optical method that can accurately measure 3D displacement by using a pair of cameras and surface speckle patterns on the object of study (IOL optics)^21^. These cameras are placed at different angles to capture images of the object from multiple viewpoints, allowing for 3D reconstruction of its surface. The speckle pattern on the surface of the object is then correlated across two synchronized images to quantify its 3D deformation. Essentially, DIC works by comparing the speckle patterns in two images and calculating how much they have shifted relative to each other, which allows for accurate measurement of the object's surface displacement^21^. It is worth noting that the use of surface speckle patterns is crucial for the success of DIC, as these patterns allow for accurate tracking of surface deformations that might not be easily visible otherwise. In ophthalmology, DIC has been employed in various studies, such as measuring displacement in sclera and cornea eye inflation tests^22,23^ and observing corneal deformation in air pulse tests, such as the Corvis ST^24^.

### Study design

Table 1 describes the seven IOLs under investigation. These lenses were purposely chosen as they cover the majority of the market for their 3 materials used (hydrophilic and hydrophobic acrylate, and PMMA) and their 6 different haptic designs, being the C-loop design (the repeated one in the study) the most common design worldwide. Each IOL was evaluated 5 times (n = 5).

**Table 1:** Properties of the IOLs under investigation. The power and optic diameter of all IOLs was + 22D and 6.00 mm. Their type clasification (flexible, mixed and stiff) was based on our previous studies^1,11^.

	IOL model (Name)	Material	Haptic design	Type	Overall size (mm)	Haptic Angle (º)	Overall design
	AcrySof IQ SN6CWS [Alcon, US] **(Acrysof IQ SN6CWS)**	Ultraviolet (UV) and blue light filtering hydrophobic acrylate	C-loop	Flexible	13.00	0	One-piece
	AKREOS AO [Bausch + Lomb, Canada] **(AKREOS AO)**	Hydrophilic acrylic (26% water content)	Four-point fixation	Mixed	10.70	0	One-piece
	AT LISA tri 839 M [ZEISS, Germany] **(AT LISA)**	Hydrophilic acrylic (25%) with hydrophobic surface properties	Plate	Stiff	11.00	0	One-piece
	AcrySof MA60BM [Alcon,US] **(Acrysof MA60BM)**	UV Acrylate/Methacrylate Copolymer (Optics) + PMMA (haptics)	Modified-C	Stiff	13.00	10	Multi-piece
	Physiol POD F GF [BVI Medical, Ireland] **(Physiol POD F GF)**	Hydrophobic acrylic	Double C	Mixed	11.40	5	One-piece
	Bi-Flex [Medicontur, Spain] **(Bi-flex)**	UV-blocking Hydrophobic acrylic	Posterior vaulting fenestrated C-loops	Flexible	13.00	0	One-piece
	Tecnis Monofocal 1-Piece [Johnson & Johnson, US] **(Tecnis)**	UV-blocking hydrophobic acrylic	C-loop	Flexible	13.00	0	One-piece

### IOL compression tests

We performed a quasi-static test to evaluate the mechanical behavior of the IOL in a wide range of compression diameter. The test consisted in compressing the IOL between two rigid clamps from a compression diameter of 11.00 up to 9.50 mm. The clamps were made of High-Density Polyethylene (HDPE) and the temperature and humidity were those of the operating room (23ºC and 28%). All IOLs were submerged in a saline solution 72 h before testing and were tested immediately after upon removal from the solution.

The two clamps were synchronously displaced at a total speed of 0.01 mm/s, which can be considered as quasi-static^25^. During the compression test, the main mechanical biomarkers of the IOL, the axial displacement, the tilt, and the rotation, were measured at a frequency of 0.25 Hz. These mechanical biomarkers are related to the visual performance of the IOL inside the eye^11^. For more information about how to obtain these mechanical biomarkers, see Fig. 3 in Cabeza-Gil et al.^11^. Additionally, 50 s more were recorded at the end of the test, when the IOLs are compressed to 9.5, to observe possible effects in the mechanical response of the IOL due to the material viscoelasticity^25^, resulting in a total test duration of 200 s (1.50 mm / 0.01 mm/s + 50 s).

### Experimental arrangement and calibration

**Figure 2:**
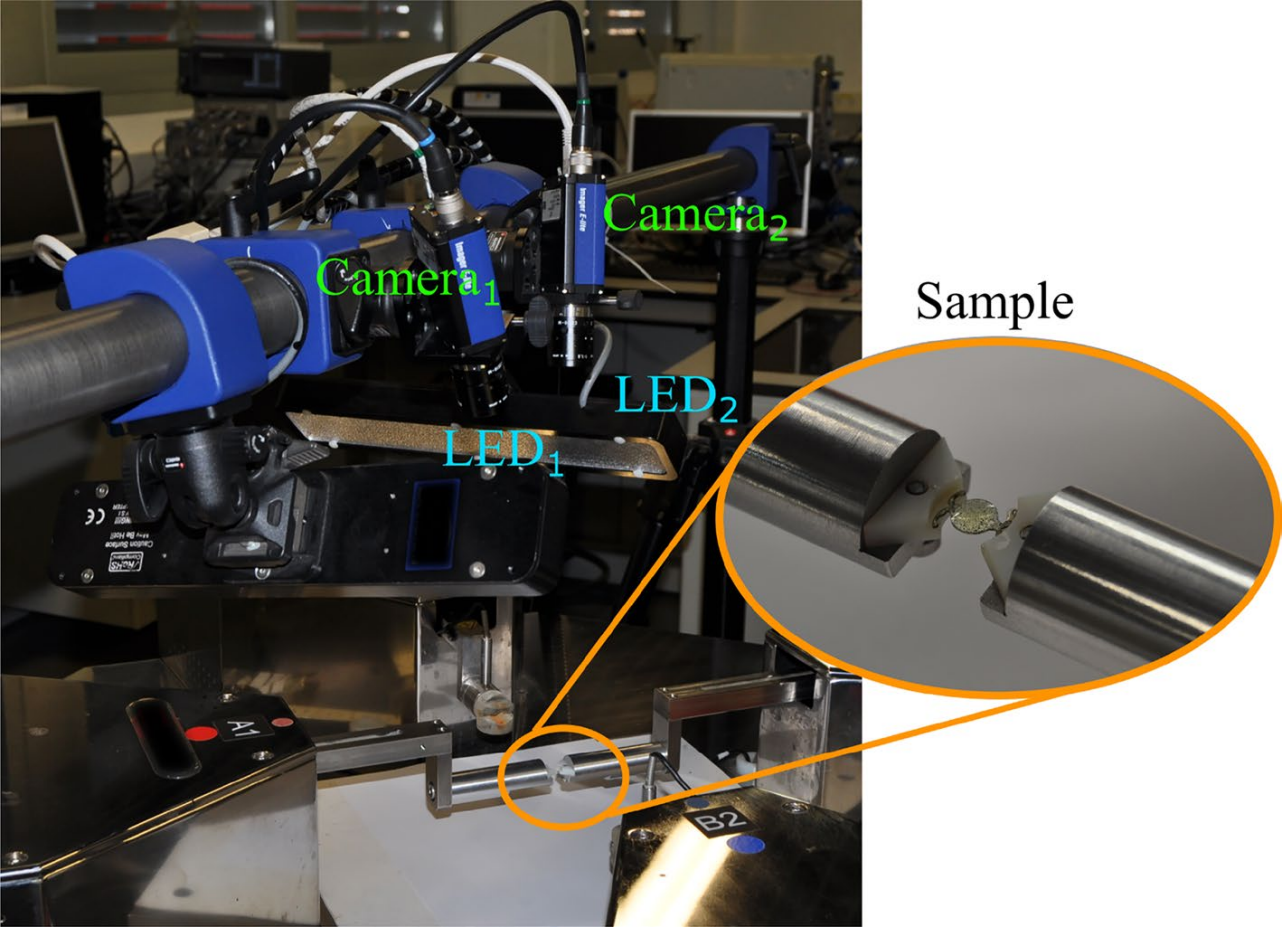
Setup of the experimental tests.

The DIC system consists of two cameras (Imager E Lite, LaVision, Germany) and a desktop computer with a Quad-core processer. The cameras have a spatial resolution of 1280 × 1024 pixels and a maximum frame rate of 500 fps. The cameras were placed at a distance of approximately 25 cm from the IOL, with a mutual distance of around 13 cm. Both cameras were mounted with an identical 200 mm f/4 lens (Nikon, Tokyo, Japan) with an opening angle of approximately 30º. The two cameras were internally synchronized with LaVision software and high-power light-emitting diodes (LEDs) fed with distensibility coefficient (DC) to avoid flickering illuminated the sample, see Fig. 2. Prior to conducting the compression tests, LaVision software performs automatic system calibration using a grid pattern.

To enable DIC to work effectively, a random pattern must be present on the specimen. For this purpose, black paint is airbrushed on top of the IOLs. The speckle pattern should have a random character, and the contrast between the speckles and the background should be as high as possible.

To evaluate the accuracy of the DIC method, a controlled axial displacement and rotation were applied to an IOL. A rotation of 5, 10 and 15º and a displacement axial of 0.10, 0.20 and 0.50 mm were applied with a controlled stepped motor. The measurements were repeated three times (n = 3) to ensure their reliability. Any IOL could have been used for this purpose since the axial displacement and rotation were induced; however, AcrySof MA60BM was specifically used.

## Data analysis

LaVision software generated 50 (0.25 images/s).vc7 files in each test. These files were processed with the PIVMat 4.20 Toolbox^26^ in MATLAB R2022a and contained both the reference coordinates and the 3D displacement (u_x_, u_y_ and u_z_) of the correlated speckle pattern (IOL optics zone).

**Figure 3:**
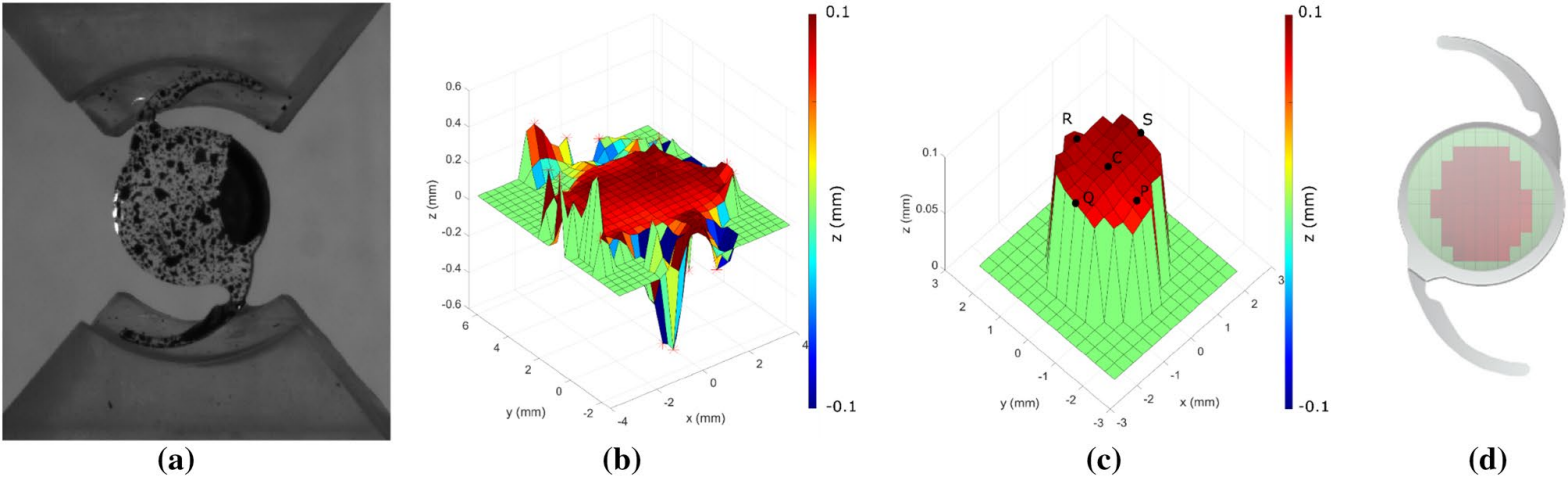
Post-processing DIC method to obtain the mechanical biomarkers of the IOL in the compression test. The speckle pattern from the IOL (**a**, **b**) is filtered to obtain the IOL optics. (**c**) C, P, Q, R, and S points are used to calculate the mechanical biomarkers. (**d**) IOL area of interest to quantify biomarkers.

Figure 3 summarizes the steps performed to process the speckle pattern data from the IOL, see Fig. 3a,b. First, the IOL optics is recognized from the speckle pattern data, see Fig. 3c. To do so, a circle of radius of 2.0 mm at the center of the speckle pattern data in Fig. 3b is used. This surface circle is assumed to be the optics of the IOL, Fig. 3d. From it, the main biomechanical biomarkers, axial displacement, tilt, and rotation, are obtained.

The axial displacement of the intraocular lens is calculated as the displacement along the axial axis of the lens from the central point (point C in Fig. 3c). To consider the possible error made when searching for the IOL optics, the axial displacement (*u*_*z*_, displacement in axis z) is calculated as the averaged value from a surface circle of 0.5 mm from the center.

The tilt and rotation are calculated considering P, Q, R, and S points following the ISO 11,979:3^10^. The optic tilt ($\Theta$) was calculated using the following equation:

$$\Theta= {\mathrm{tan}}^{-1}\sqrt{{{s}_{1}}^{2}+{{s}_{2}}^{2}}$$

where slopes s_1_ and s_2_ are calculated as $\frac{PRy}{PRx}$ and $\frac{QSy}{QSx}$, being PR_x,y_ and QS_x,y_ the relative distance between points (P,Q,R,S) in the x- or -y axis, respectively.

Rotation is calculated as the angle difference between the PR_x_ vector in the horizontal plane at the deformed and reference state. The IOL optics can resemble a solid rigid during the compression test since the part of the IOL that deforms are the haptics^27^. Therefore, the possible error in the tilt and rotation calculation from this assumption is minimal.

Some tests were discarded due to the non-recognition of the speckle pattern by LaVision software.

## Results

### Accuracy and precision of the DIC method in IOLs

**Figure 4:**
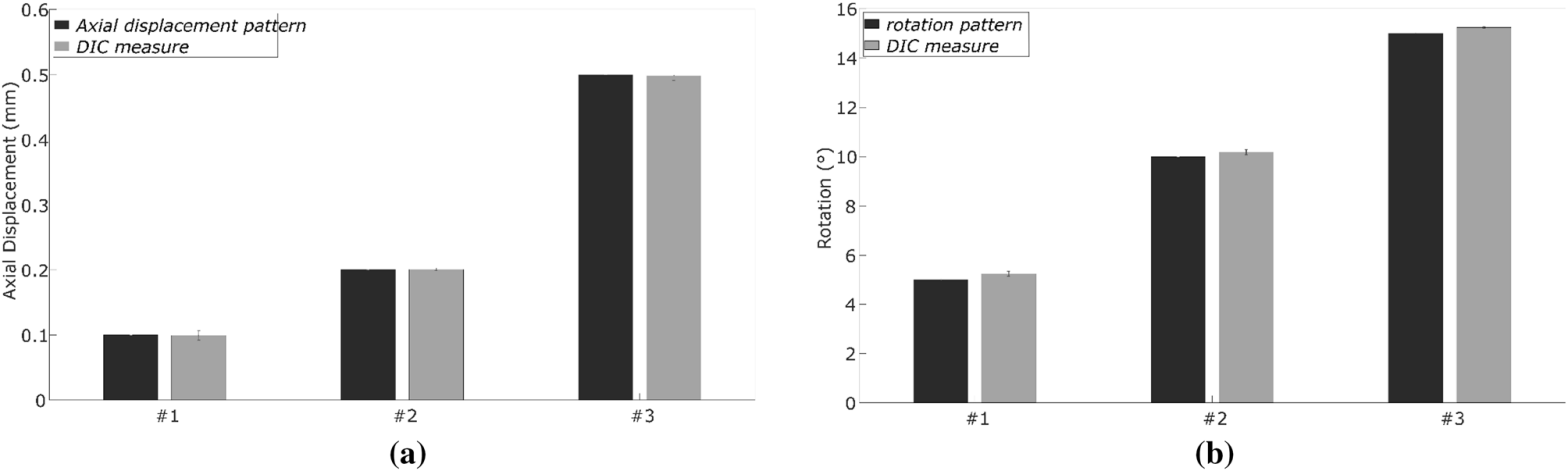
Control tests. (**a**) Axial displacement. (**b**) Rotation.

The accuracy of the IOL axial displacement and the rotation are reflected in Fig. 4. For the imposed 0.10 mm of axial displacement, DIC methodology ranged from 9.17·10^–2^ to 10.34·10^–2^ mm (mean ± std = 9.34·10^–2^ ± 0.70·10^–2^ mm). For the imposed 0.20 mm of axial displacement, DIC methodology ranged from 1.99·10^–1^ to 2.01·10^–1^ mm (mean ± std = 2.00·10^–1^ ± 0.21·10^–2^ mm). For the imposed 0.50 mm of axial displacement, DIC methodology ranged from 4.93·10^–1^ to 5.07·10^–1^ mm (mean ± std = 4.98·10^–1^ ± 0.73·10^–2^ mm).

For the 5º imposed rotation, the DIC method ranged from 5.11º to 5.32º (mean ± std = 5.20 ± 0.12º). For the 10º imposed rotation, the DIC ranged from 10.16 to 10.31º (mean ± std = 10.21 ± 0.13º), whilst for the 15º imposed rotation, the DIC ranged from 15.22 to 15.23º (mean ± std = 15.23 ± 0.07º). The level of precision of DIC method for IOLs was calculated as the variability range obtained in the control tests, 1.17·10^–2^ mm for IOL axial displacement and 0.20º for rotation.

### Mechanical behavior of the IOLs under investigation

**Figure 5:**
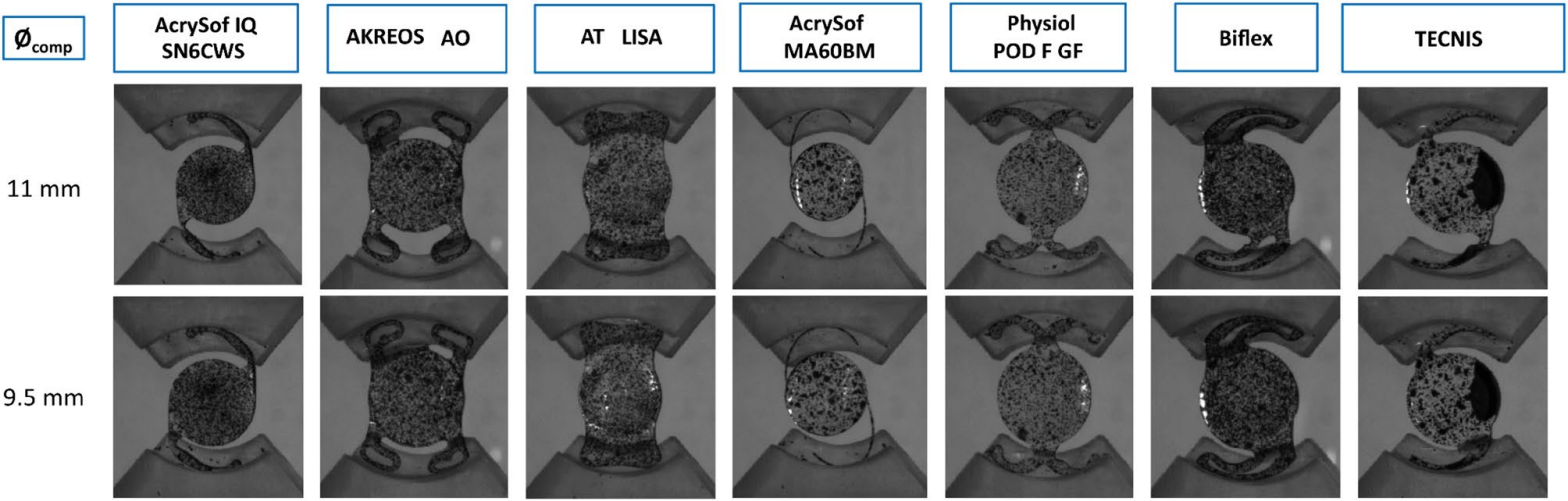
Plan view of the reference *(ø*_*comp*_ = 11.00 mm) and deformed state (ø_comp_ = 9.50 mm) of the seven IOLs under investigation.

Figure 5 shows the plan view of the reference (Ø = 11.00 mm) and deformed state (Ø = 9.50 mm) of the seven IOLs analyzed. Most of the IOL designs presented a small axial displacement (< 0.10 mm) at a compression diameter of 9.50 mm, except for AT LISA and Acrysof MA60BM models, which presented an axial displacement of 1.36 ± 0.20 mm and 0.36 ± 0.82 mm and a tilt of 5.89 ± 2.55º and 8.50 ± 8.45º, respectively. These values are clinically significant, i.e., affecting the patient's visual quality^11,28^.

**Figure 6:**
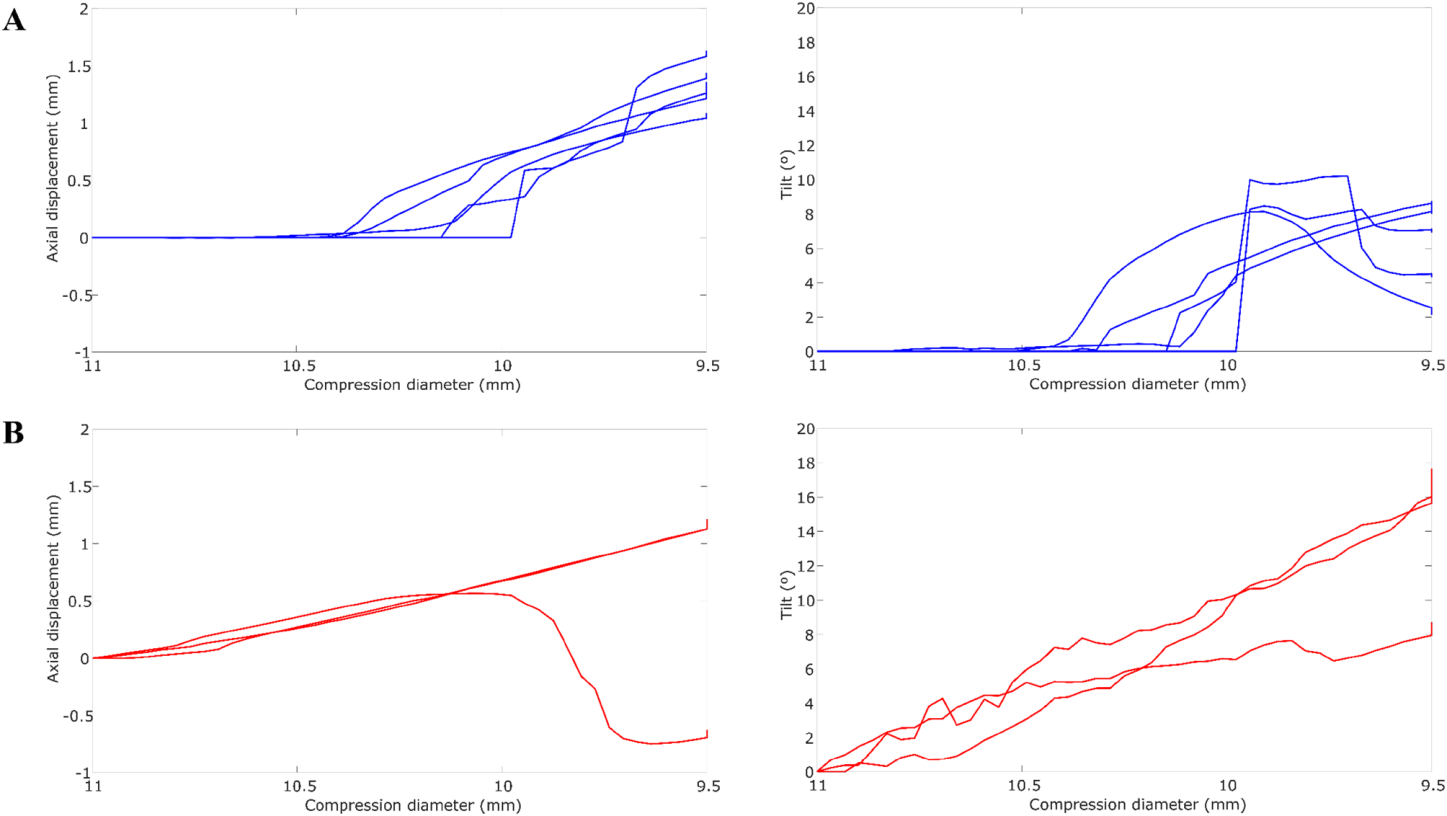
Axial displacement and tilt in the compression tests of the (**A**) AT LISA and (**B**) Acrysof MA60BM IOL models.

Figure 6 shows the axial displacement and tilt of the stiff IOL designs (AT LISA and Acrysof MA60BM models) through the compression diameter range [11.00–9.50 mm]. AT LISA and Acrysof MA60BM models are stiff designs that probably do not work under those compression levels (lower than 10.50 mm), as they are prone to largely deform the capsule bag^29^. For compression diameters larger than 10.50 mm, AT LISA barely presented axial displacement (0.00 ± 0.01 mm), tilt (0.03 ± 0.01º), and rotation (0.15 ± 0.08º).

**Figure 7:**
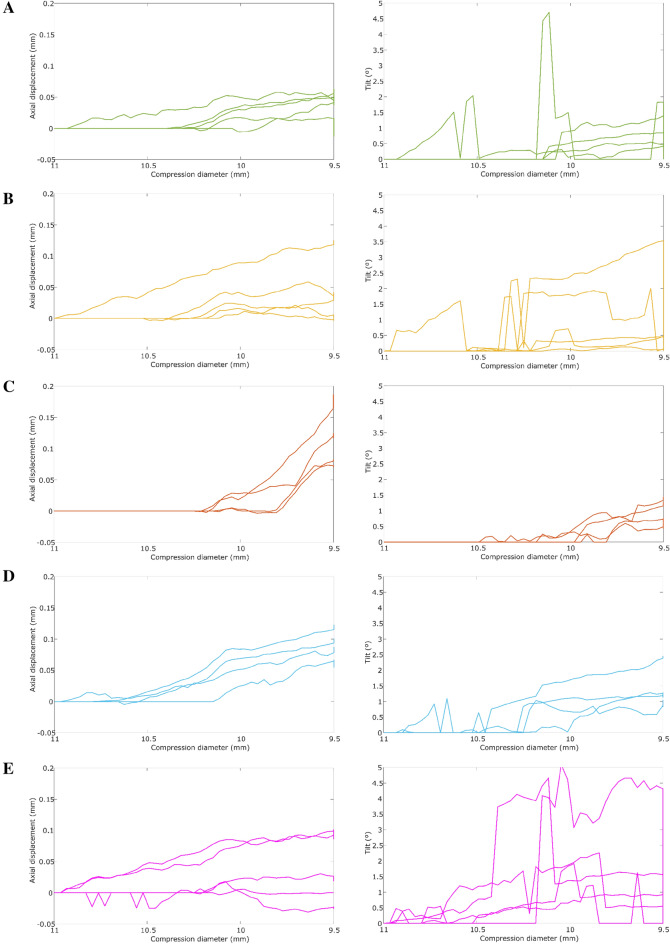
Axial displacement and tilt in the compression tests of the following IOL models: (**A**) AcrySof IQ SN6CWS, (**B**) Akreos AO, (**C**) Physiol POD F GF, (**D**) Bi-Flex and (**E**) Tecnis.

Figure 7 shows the axial displacement and tilt of the flexible and mixed IOL designs (AcrySof IQ SN6CWS, AKREOS AO, Physiol POD F GF, Bi-Flex and Tecnis) through the compression diameter range [11.00–9.50 mm]. The mechanical behavior of AcrySof IQ SN6CWS and AKREOS AO models was very similar, barely presenting axial displacement, tilt, and rotation. The Tecnis model presented also a similar behavior, except for the tilt, whose value might have a little clinical relevance (1.84º ± 1.32º for Ø = 9.50 mm)^11,28^. We analyzed in a previous study that that an axial displacement lower than 0.1 mm and tilt lower than 2.0º might not have a clinical relevance, although this also depends on the introduced optical design^11^.

Physiol POD F GF model also barely presented axial displacement up to a compression diameter of 9.70 mm, from where it starts to increase its axial displacement exponentially (Fig. 7c). Physiol POD F GF model presented an adequate mechanical stability before reaching the critical compression diameter (Ø = 9.70 mm) as the mechanical biomarkers were minimal until this compression diameter range.

Biflex model presented a slightly higher axial displacement than AcrySof IQ SN6CWS, AKREOS AO and Tecnis, but those values have little clinical relevance. Table 2 summarizes the mechanical biomarkers for the seven IOLs under investigation at a compression diameter of 10.50, 10.00 and 9.50 mm.

**Table 2:** Mean and standard deviation (n = 5) of the 7 IOLs under investigation for a compression diameter of 10.50, 10.00 and 9.50 mm. Values for the compression diameter of 9.5 are at the end of the test.

	IOL model	Axial displacement (mm)			Tilt (º)			Rotation (º)		
	*ø*_*comp*_	*10.50*	*10.00*	*9.50*	*10.50*	*10.00*	*9.50*	*10.50*	*10.00*	*9.50*
	AcrySof IQ SN6CWS [Alcon, US]	0.01 ± 0.01	0.03 ± 0.02	0.04 ± 0.03	0.41 ± 0.80	0.64 ± 0.50	0.69 ± 0.49	0.85 ± 0.17	1.48 ± 0.36	2.48 ± 0.72
	AKREOS AO [Bausch + Lomb, Canada]	0.01 ± 0.01	0.03 ± 0.03	0.04 ± 0.04	0.02 ± 0.05	1.05 ± 0.87	0.28 ± 0.22	0.25 ± 0.37	0.63 ± 0.53	1.61 ± 0.31
	AT LISA tri 839 M [ZEISS, Germany]	0.00 ± 0.01	0.44 ± 0.26	1.37 ± 0.18	0.03 ± 0.01	3.87 ± 2.47	5.89 ± 2.23	0.15 ± 0.08	0.17 ± 0.02	0.69 ± 0.17
	AcrySof MA60BM [Alcon,US]	0.17 ± 0.14	0.38 ± 0.30	0.36 ± 0.72	2.51 ± 2.18	5.15 ± 4.26	8.50 ± 7.41	0.93 ± 0.21	2.08 ± 0.44	3.62 ± 1.19
	Physiol POD F GF [BVI Medical, Ireland]	0.00 ± 0 .00	0.01 ± 0.01	0.09 ± 0.06	0.00 ± 0.00	0.12 ± 0.13	0.76 ± 0.50	0.11 ± 0.08	0.17 ± 0.06	0.10 ± 0.10
	Bi-Flex [Medicontur, Spain]	0.00 ± 0.01	0.04 ± 0.03	0.07 ± 0.04	0.04 ± 0.05	0.74 ± 0.58	1.31 ± 0.57	0.32 ± 0.26	0.61 ± 0.44	1.11 ± 0.66
	Tecnis Monofocal 1-Piece [J&J, US]	0.02 ± 0.02	0.04 ± 0.03	0.03 ± 0.04	0.39 ± 0.40	2.06 ± 1.32	1.84 ± 1.32	0.44 ± 0.15	0.92 ± 0.23	1.58 ± 0.37

### IOL repositioning (viscoelastic effect)

**Figure 8:**
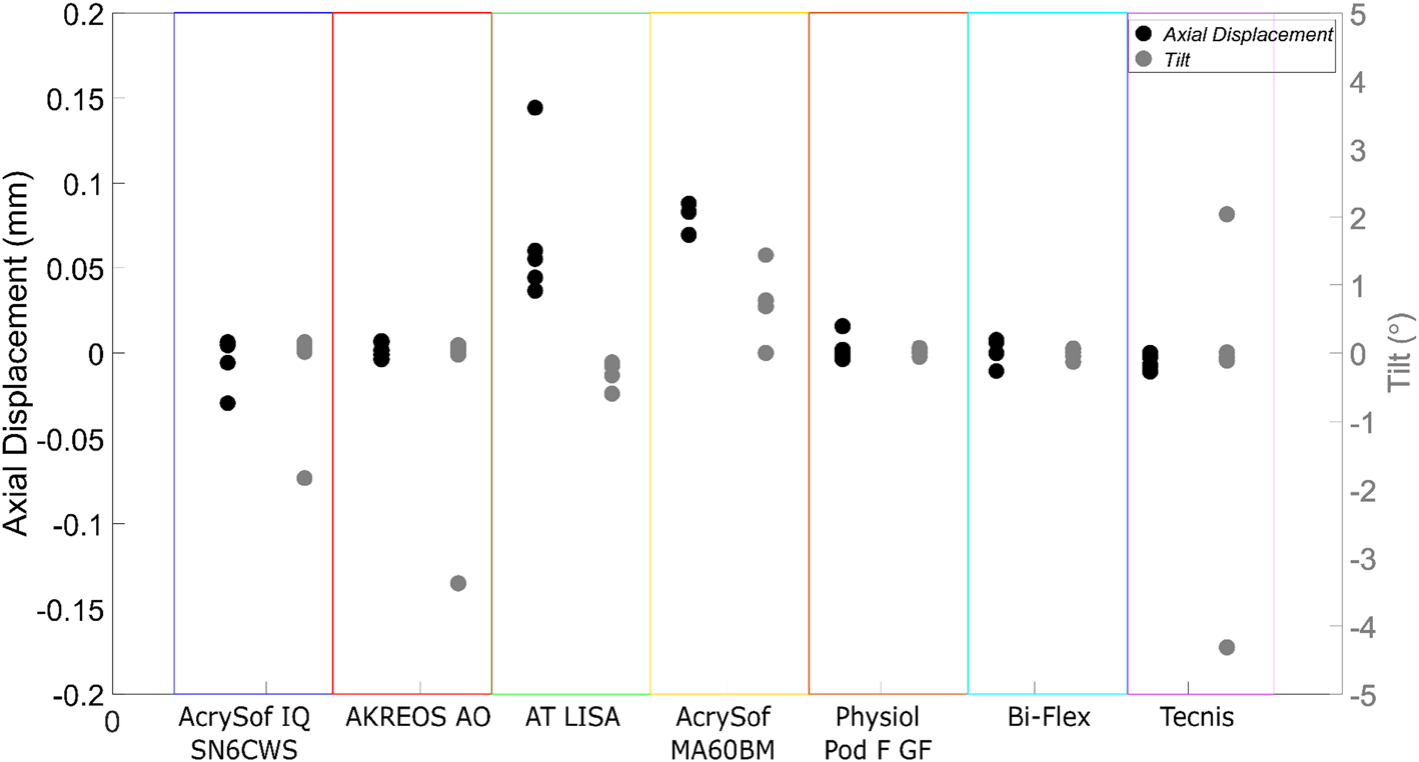
Change in the axial displacement and tilt (mean and std) during the last 50 s of the tests where the IOLs are compressed at 9.5 mm.

Figure 8 shows the change in the axial displacement and tilt for the seven IOLs under investigation at the end of the test, i.e., after 50 s after compressing until Ø = 9.50 mm. A mean axial displacement change of 0.01 ± 0.04 mm and a mean tilt change of 0.33 ± 0.21º was observed for the seven IOLs. Overall, a repositioning was noted for AT LISA (an axial displacement change of (0.06 ± 0.04 mm) and AcrySof MA60MB (an axial displacement change of (0.05 ± 0.04 mm). The other IOLs did not undergo repositioning, except for one exceptional case in AcrySof IQ SN6CWS, AKREOS AO and Tecnis models were the IOL tilt was restored to 0º.

### IOL behavior depending on IOL material and haptic design

**Figure 9:**
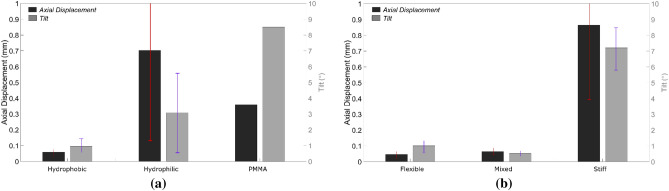
Axial displacement and tilt (mean and std) at the compression diameter of 9.50 mm according to the IOL material (**a**) and haptic design (**b**). The haptic design classification was divided into flexible (AcrySof IQ SN6CWS, Bi-Flex and Tecnis), mixed (Akreos AO and Physiol POD F GF) and stiff designs (AT LISA and AcrySof MA60BM).

Figure 9 presents the axial displacement and tilt at Ø = 9.50 mm according to the IOL material (See Table 1) and haptic design (flexible, mixed and stiff). A mean and standard deviation axial displacement of 0.05 ± 0.02 mm, 0.70 ± 0.66 mm , and 0.36 mm were obtained for the hydrophobic IOLs, hydrophilic IOLs, and the PMMA IOL. A tilt of 0.93 ± 0.24º, 3.08 ± 0.80º , and 8.50º were obtained for the same groups, respectively. Regarding the haptic design classification, an axial displacement of 0.04 ± 0.01 mm, 0.06 ± 0.02 mm , and 0.86 ± 0.50 mm and a tilt of 0.99 ± 0.25º, 0.51 ± 0.02º , and 7.15 ± 1.30º were obtained for the flexible, mixed and stiff IOL designs. No statistically significant difference of the axial displacement and tilt was found between any of the material groups (*p* > 0.05) whilst a statistically significant different was found between stiff and the others depending on the classification haptic design.

## Discussion

This study aimed both to analyze the mechanical stability of seven IOLs with different haptic designs and to provide a method to automatically quantify the IOL mechanical properties under quasi-static compression (Ø [11.00–9.50 mm]) using DIC. DIC avoids the use of manual measurement (For example, images containing a scale^11^) and might increase the accuracy. The accuracy and reliability of the method was calculated through control tests, which showed an accuracy higher than 0.01 mm and a precision around 1.17·10^–2^ mm for axial displacement. For rotation, an accuracy and precision of 0.1º mm and around 0.2º, respectively, was obtained.

C-loop IOL designs (AcrySof IQ SN6CWS, AKREOS AO, Tecnis, Biflex) presented the best mechanical response for a compression diameter (Ø) of 9.50 mm as these IOLs are intended to adapt to the capsular bag shape, comparing with the stiff designs (AT LISA and Acrysof MA60BM), which largely deform the bag^29^. Tecnis model presented a clinically significant tilt, probably caused by the short length of its haptics, which provide a less haptic-contact surface^30^. The response of Biflex IOL was slightly worse as its design can be considered as mixed between flexible and stiff, as POD F GF model.

The mechanical behavior obtained for the AcrySof SN6CWS model was similar to those obtained in the literature^4,12^, barely presenting axial displacement, tilt and rotation for all the compression diameter range (Table 2). For the Tecnis IOL, we obtained a lower axial displacement (0.04 ± 0.03 mm) and higher tilt (2.06 ± 1.32 º) for a compression diameter of 10.00 mm than Lane et al.^4^ (0.14 ± 0.02 mm) and (0.7 ± 0.4 º), respectively, and similar results to Bozukova et al.^12^ for the axial displacement (0.03 mm) and tilt (0.64º). The difference across studies might be related to the manual measurement variability. We obtained a similar axial displacement for AcrySoft MA60BM at Ø = 9.50 mm (1.21, 1.20 and -0.62 in the three tests performed) than a similar multi-piece IOL, the Sensar AR40e model (Johnson & Johnson, US)^12^, which resulted in 1.13 mm. We observed that the IOL can moved axially in both -z directions. However, for a 10.00 mm compression diameter we obtained 0.67, 0.66 and 0.56 mm against 0.20 mm^12^. Observing the fluctuating mechanical behavior of this IOL, this could be produced due to the well diameter variability.

POD F GF results are similar to those obtained in literature^12^, obtaining an axial displacement and tilt of 0.09 ± 0.06 mm and 0.76 ± 0.50º against 0.09 mm and 1.74º for a compression diameter of 9.50 mm. Similar results were obtained for the other compression diameters. The results obtained are also comparable to in silico values (0.09 ± 0.06 mm against 0.03 mm for Ø = 9.50 mm), providing adequate mechanical stability for all the compression diameter range tested [11.00 to 9.50 mm]^31^.

Andreas and Eva-Maria Borkenstein^30^ recently evaluated the haptic-geometry response of five different C-loop IOLs using computed tomography for 11.50, 11.00, 10.00 and 9.00 mm compression diameters. Although they observed a different mechanical response depending on the optic-haptic junction characteristics of the IOLs, they did not quantify the mechanical biomarkers, making difficult the comparison across studies.

One limitation of compressing the IOL in a well according to ISO 11,979:3 or in a quasi-static test between two clamps is that the rotation or decentration are not reliable measures since the test conditions are not similar to those in vivo. The IOL might rotate in vivo because of mechanical stability, surgeon placement, fusion footprint postcapsular bag shrinkage, etc., situations that do not reproduce the IOL compressing between two clamps. One alternative to measure this outcome is the inclusion of in vitro models, which can provide valuable outputs of the overall IOL response in time^15^. On the other hand, we previously demonstrated numerically that the axial displacement and tilt values can be reliable at the same IOL diameter compression in the tests as in vivo^29^.

## Conclusions

The study highlights the capability of combining quasi-static compression testing with Digital Image Correlation to accurately and automatically quantify the mechanical biomarkers of intraocular lenses (IOLs) across a wide range of compression diameters. Specifically, the method proves effective in identifying the critical compression diameter, where the response of the IOL may become unstable, and detect different mechanical behavior depending on the IOL classification.

## Data Availability

The datasets used and/or analyzed during the current study available from the corresponding author on reasonable request.
